# Cortical correlates in upright dynamic and static balance in the elderly

**DOI:** 10.1038/s41598-021-93556-3

**Published:** 2021-07-08

**Authors:** Maria Rubega, Emanuela Formaggio, Roberto Di Marco, Margherita Bertuccelli, Stefano Tortora, Emanuele Menegatti, Manuela Cattelan, Paolo Bonato, Stefano Masiero, Alessandra Del Felice

**Affiliations:** 1 Department of Neuroscience, Section of Rehabilitation, University of Padua, Padova, 35128 Italy; 2 Department of Information Engineering, University of Padua, Padova, Italy 35131; 3 Department of Statistical Sciences, University of Padua, Padova, 35121 Italy; 4 Department of Physical Medicine and Rehabilitation, Harvard Medical School, Spaulding Rehabilitation Hospital, Charlestown, Boston, MA 02129 USA; 5 Wyss Institute for Biologically Inspired Engineering, Harvard University, Boston, MA 02115 USA; 6 Padova Neuroscience Center, Padova, 35128 Italy

**Keywords:** Neuroscience, Motor control, Motor cortex, Biomedical engineering

## Abstract

Falls are the second most frequent cause of injury in the elderly. Physiological processes associated with aging affect the elderly’s ability to respond to unexpected balance perturbations, leading to increased fall risk. Every year, approximately 30% of adults, 65 years and older, experiences at least one fall. Investigating the neurophysiological mechanisms underlying the control of static and dynamic balance in the elderly is an emerging research area. The study aimed to identify cortical and muscular correlates during static and dynamic balance tests in a cohort of young and old healthy adults. We recorded cortical and muscular activity in nine elderly and eight younger healthy participants during an upright stance task in static and dynamic (core board) conditions. To simulate real-life dual-task postural control conditions, the second set of experiments incorporated an oddball visual task. We observed higher electroencephalographic (EEG) delta rhythm over the anterior cortex in the elderly and more diffused fast rhythms (i.e., alpha, beta, gamma) in younger participants during the static balance tests. When adding a visual oddball, the elderly displayed an increase in theta activation over the sensorimotor and occipital cortices. During the dynamic balance tests, the elderly showed the recruitment of sensorimotor areas and increased muscle activity level, suggesting a preferential motor strategy for postural control. This strategy was even more prominent during the oddball task. Younger participants showed reduced cortical and muscular activity compared to the elderly, with the noteworthy difference of a preferential activation of occipital areas that increased during the oddball task. These results support the hypothesis that different strategies are used by the elderly compared to younger adults during postural tasks, particularly when postural and cognitive tasks are combined. The knowledge gained in this study could inform the development of age-specific rehabilitative and assistive interventions.

## Introduction

A combination of risk factors that increase with age triggers falls in the elderly. The most significant number of fatal falls each year are reported in old adults^1^.

In a normally functioning nervous system, maintaining an upright stance is predominantly controlled by cortical and spinal networks. Cortical control is challenged by cognitive loads in different ways, depending on the postural task, the individuals’ age, and their balance abilities^2^. Postural responses are likely influenced by the cerebral cortex directly via corticospinal loops and indirectly via communication with the brainstem centers harboring the interactions for postural responses, providing both speed and flexibility for a suitable reaction to a loss of balance^3^.

Complex balance tasks using different kinds of perturbations (e.g., either a platform perturbation or a cognitive task, namely a dual-task) have been assessed via computerized dynamic posturography (CDP), which consists of measuring and analyzing the center of pressure time series during various balancing tasks (e.g., quiet standing, standing on an unstable surface, standing while performing cognitive tasks)^4^. CDP provided evidence that the elderly prioritize balance recovery when performing a cognitive task while responding to a balance perturbation. CDP was also performed in virtual-reality environments, where visual interference led to an increase in postural sway^2,5^. Other studies exploring the relationship among attention, posture, and gait in younger and older adults proved that postural control is more cognitively demanding in the elderly than in younger adults. Their balance is more severely affected while double tasking^6–13^. It is unclear, however, if these differences are due to split attention between the two tasks, reduced attention ability, or increasing demand due to impairments affecting the postural control system in the aging nervous system^14^.

Assessing the neurophysiological characteristics of the control of balance in increasingly challenging tasks (static and dynamic balance, with/without a cognitive task) may help to clarify age-related differences. Our focus was on dual-task experiments^15^, often designed to simulate real-life situations of instability. In young, healthy volunteers walking while conversing or texting, electroencephalographic (EEG) data showed an activation of the posterior parietal cortex (PPC)^15^ which seems to integrate afferent inputs to facilitate changes in motor strategy during walking^15–17^. When younger adults walked without engaging in any additional tasks, the PPC displayed activity in the theta range (4–7 Hz). When a dual-task was added, a relationship could be identified between PPC activity in the alpha frequency band and walking pace: region- and frequency- specific brain activation patterns could predict gait stability during dual-task walking through a multiple regression model^15^. Vertical trunk acceleration was predicted by gait velocity and left PPC theta (4–7 Hz) frequency band power in single-task walking and by gait velocity and left PPC alpha (8–12 Hz) frequency band power in walking while conversing. Medio-lateral trunk acceleration was predicted by left PPC beta (15–25 Hz) frequency band power when walking and texting. Region- and frequency- specific brain activation patterns were also found to coordinate navigation performance^18,19^. The existence of neural markers of postural instability that may trigger balance compensatory adjustments was also supported by the observation that a burst of gamma activity preceded the initiation of compensatory backward posture adjustments^20^.

Despite their potential relevance in the above-described context, there is a lack of studies investigating the EEG and electromyographic (EMG) data in the elderly during a balance task^6^. The few studies addressing this issue^21,22^ showed that scalp EEG power distribution increases in elderly and young adults in the delta frequency band during challenging postural conditions. Theta rhythms were more responsive to cognitive tasks particularly in younger subjects. Gamma oscillations increased in the elderly primarily over the central and centro-parietal cortices during challenging postural tasks.

Most studies analyzing cortical oscillations power spectrum density changes associated with postural control are based on younger adults^14^. Beta cortico-muscular coherence in young adults was responsive to mechanical changes, but not to visual or surface changes, during standing balance tests^23^. An increase in frontal and parietal midline theta power was observed in association with demanding balance control tasks^24^. Changes in fronto–central and centro–parietal theta power were associated with balance performance^24^. Clusters of EEG sources obtained with Independent Component Analysis (ICA) in healthy young subjects walking heel-to-toe on a treadmill-mounted balance beam and walking on the treadmill belt at the same speed identified activations in or near the anterior cingulate, anterior parietal, superior dorsolateral-prefrontal, and medial sensorimotor cortex. These areas exhibited significantly larger mean spectral power in the theta band (4–7 Hz) during walking on the balance beam compared with treadmill walking. Spectrogram-based analyses demonstrated that the first electrocortical indication of impending loss of balance occurred in the left sensorimotor cortex at the transition from single support to double support before stepping off the beam^17^. In people who trained extensively to improve their balance control, such as athletes, the greater the alpha event-related desynchronization was, the more significant the reduction in sway area was^25^.

Despite large heterogeneity in experiments, protocols, and data analyses^6^, findings based on EMG data are more consistent, though their interpretation is still controversial. EMG latency, amplitude and muscular co-contractions are usually more prominent in the elderly during walking and upright stance tasks^26–31^. Increased activation of antagonistic muscles appears to be associated with healthy aging in walking tasks^32^, and with balance impairments in balance recovery^33^.

Our study aimed to investigate cortical and muscular activation patterns underlying age-related differences in postural control. Co-recording EEG and EMG data in the dual-task condition in healthy subjects mimics a real-life situation of distraction that might lead to balance instability in the elderly. We chose a visual task because visual and proprioceptive inputs appeared to play dominant roles in assuring postural stability^5^.

Considering the reported heterogeneous findings, our research questions are: (Q1) Which are the age-related differences in cortical and muscular activations during static and dynamic balance? (Q2) How do these differences change during dual postural-visual tasks?

## Results

### Behavioral tests

**Figure 1:**
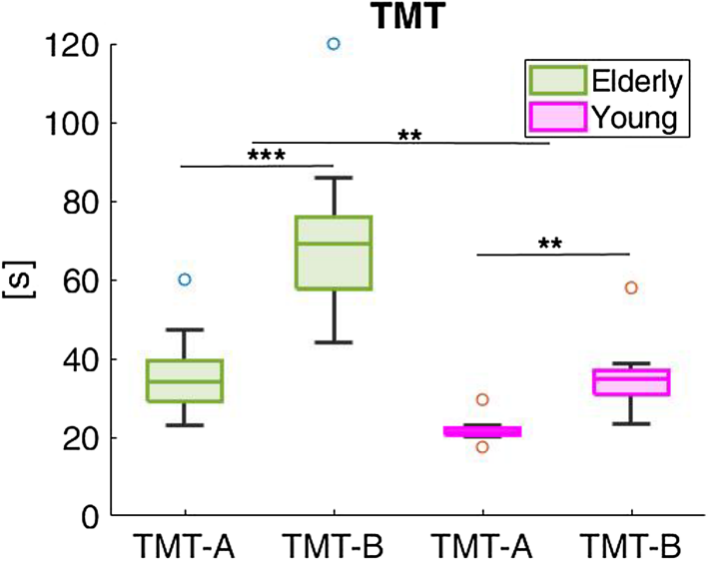
Time in seconds to perform the Trail Making Test (TMT) A and B for the elderly (in green) and for the younger adults (in pink). On each box, the central line indicates the median, and the bottom and top edges of the box indicate the 25th and 75th percentiles, respectively. Extreme data points–not considered outliers – are plotted individually using the ’o’ symbol. Asterisks indicate statistical significant difference (’**’ stands for *P*_value_ < 0.01; ’***’ stands for *P*_value_ < 0.001).

We administered the Trail Making Test (TMT) A and B as a screening tool to detect visual-attention and movement speed impairments, common with aging. These deficits were considered as exclusion criteria , i.e., participants performing under the threshold of normality, based on normative values computed by taking into account the subject’s age and education level^34^ were excluded. The raw time in *s* for TMT-A (assessing cognitive processing speed) and TMT-B (assessing executive functioning) is reported in Fig.  1 and in Table 1 (2nd and 4th columns). Since performance on TMT-A and TMT-B is affected by age and education^35^, we computed the cut-off for each participant considering these two covariates^36^ (3rd and 5th columns of Table 1). None of our participants showed an impaired performance considering their age and level of education, comparing the time needed to perform the tasks (2nd and 4th columns of Table 1) and the cut-offs (3rd and 5th columns of Table 1). The time required to complete the tests was normally distributed for both groups, and significantly higher in the TMT-B vs. TMT-A execution in particular for the elderly (*P*_value_ < 0.001) and in both tests in the elderly compared to the younger adults (*P*_value_ < 0.01). TMT performance declines, on average, during healthy aging^34^, with performance on TMT-B declining significantly more than TMT-A in older adults^37^.

**Table 1:** TMT-A and TMT-B: results.

Subject	TMT-A	Cut-off (TMT-A)	TMT-B	Cut-off (TMT-B)
Young adults				
CTRL002	21.24	45	38.66	120
CTRL003	13.88	45	21.00	120
CTRL004	21.36	45	23.30	120
CTRL005	17.50	45	28.00	120
CTRL006	20.01	45	57.97	120
CTRL007	22.95	45	35.32	140
CTRL008	29.56	45	34.43	120
CTRL009	22.05	45	34.91	140
Elderly				
ELD001	34.04	68	60.07	200
ELD002	25.30	68	44.20	200
ELD003	37.00	68	86.00	200
ELD004	60.00	85	120.00	220
ELD005	23.00	68	51.00	200
ELD006	36.10	68	69.04	200
ELD007	30.58	68	69.85	200
ELD008	31.99	85	72.61	220
ELD009	47.22	68	62.44	200

### Balance control

**Figure 2:**
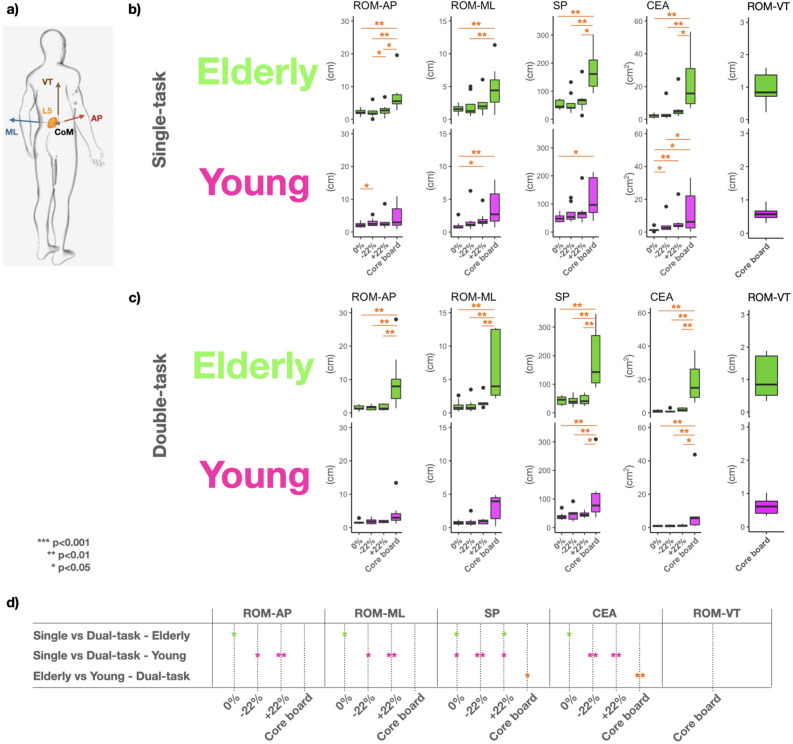
(**a**) Sensor location and adjusted sensitive axes: anterior-posterior (AP), medial-lateral (ML) and vertical (VT) direction of the center-of-mass (CoM) trajectory, as estimated via the sensor placed on the fifth lumbar vertebra (L5). Balance control performances during static (0%, ± 22%) and dynamic postural (Core board) tasks in (**b**) single-task and (**c**) dual-task for elderly and younger adults. d) Within-group statistical differences (i.e., single- vs. dual-task) in each postural condition for elderly and younger adults, and between-group differences (i.e., elderly vs. young) in each postural condition in dual-task conditions. No significant differences were detected for single-task in between-group comparisons. Statistical differences among conditions are highlighted in panel b) and c). Variables: Range of Motion (ROM), Sway Path length (SP), 95% Confidence Ellipse Area (CEA).

Visual inspection of the results in Fig. 2b and c, shows all variables with larger sway and variability in the elderly in single- and dual-tasking. Statistically significant differences (Fig. 2d) were obtained for the Sway Path length (SP) (*P* < 0.05) and the Confidence Ellipse Area (CEA) during the dynamic postural task with dual-tasking (*P* < 0.01).

Within-group comparisons (i.e., single- vs. dual-task) showed a significant difference for the Range of Motion in the antero-posterior and medio-lateral directions (ROM-AP and ROM-ML), for the Sway Path length (SP) and Confidence Ellipse Area (CEA) in the 0% condition, and for the SP in the + 22% condition in the elderly (*P* < 0.05) (Fig. 2d, first row). In the younger adults (Fig. 2d, second row), all variables differed in − 22% (ROM-AP and ROM-ML: *P* < 0.05; SP and CEA: *P* < 0.01) and + 22% (SP: *P* < 0.05; ROM-AP, ROM-ML and CEA: *P* < 0.01), and in the SP in the 0% condition (*P* < 0.05). Significant differences for between-groups comparison (i.e., elderly vs. younger adults in single-task, and elderly vs. younger adults in dual-task) were only obtained in the core board condition and in dual-task (SP: *P* < 0.05; CEA: *P* < 0.01; Fig. 2d, third row).

Cortical activation was estimated with power spectra of the brain cortex parcelled in 45 regions of interest (ROIs). The power spectral density in each region of interest followed the 1/f power law pattern. All comparisons of the power spectra in the same ROI among the estimated frequency bands resulted statistically significant. Results during single-task (Q1) and dual-task (Q2) conditions are reported in the following paragraphs.

### Cortical activation underlying age-related differences in postural control (Q1)

**Figure 3:**
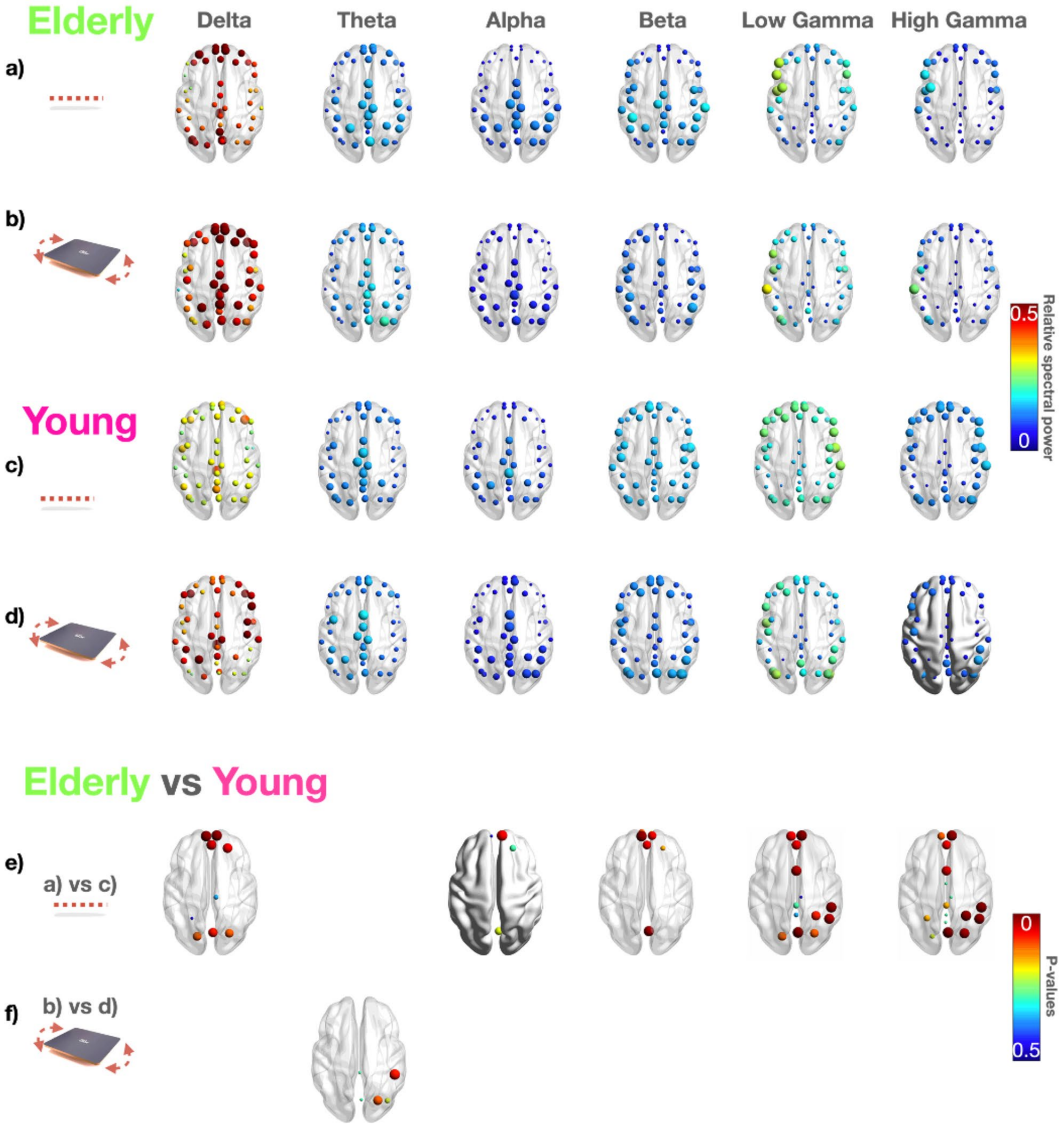
EEG power spectra during static (0%) and dynamic balance task (core board). Median values computed among elderly in (**a**) and (**b**) and among younger adults in (**c**) and (**d**) for each ROI represented by a sphere centered on the cortical region, whose radius is linearly related to the EEG power spectrum magnitude. Such information for panels (**a**–**d**) is also coded through a color scale titled “Relative power spectra”. In particular, we reported the power spectra values for each EEG frequency band (ordered per columns) during static balance (0%) in (**a**) and (**c**) and during dynamic balance on the core board in (**b**) and (**d**). The *P*-values showing statistically significant differences between the elderly and the younger adults were reported in panel (**e**) during the static balance test (0%) and (**f**) during the dynamic balance test (core board). Each *P* < 0.05 is represented by a sphere centered on the cortical region, whose radius is linearly related to the magnitude, also coded through a color scale titled “*P*-values”.

Figure 3 reports cortical activations in the two groups during static and dynamic balance tests.

During static balance (i.e., undisturbed upright standing), the elderly (Figure 3a) exhibit higher values in the low frequency rhythms (delta); the younger adults show higher values in higher frequency rhythms (i.e., beta, low and high gamma) over the frontal and midline regions (Fig. 3c). Significant differences (*P* < 0.05) between the two groups were identified over frontal regions in the delta band (higher values in the elderly) and over frontal and sensorimotor areas in the beta and gamma bands (higher values in younger adults).

During dynamic balance tasks (i.e., on the core board), the theta rhythm over the motor/occipital area displayed an increase in the elderly (Fig. 3b,d) (Wilcoxon test (Fig. 3f).

### Cortical activations underlying age-related differences during dual postural-visual tasks (Q2)

**Figure 4:**
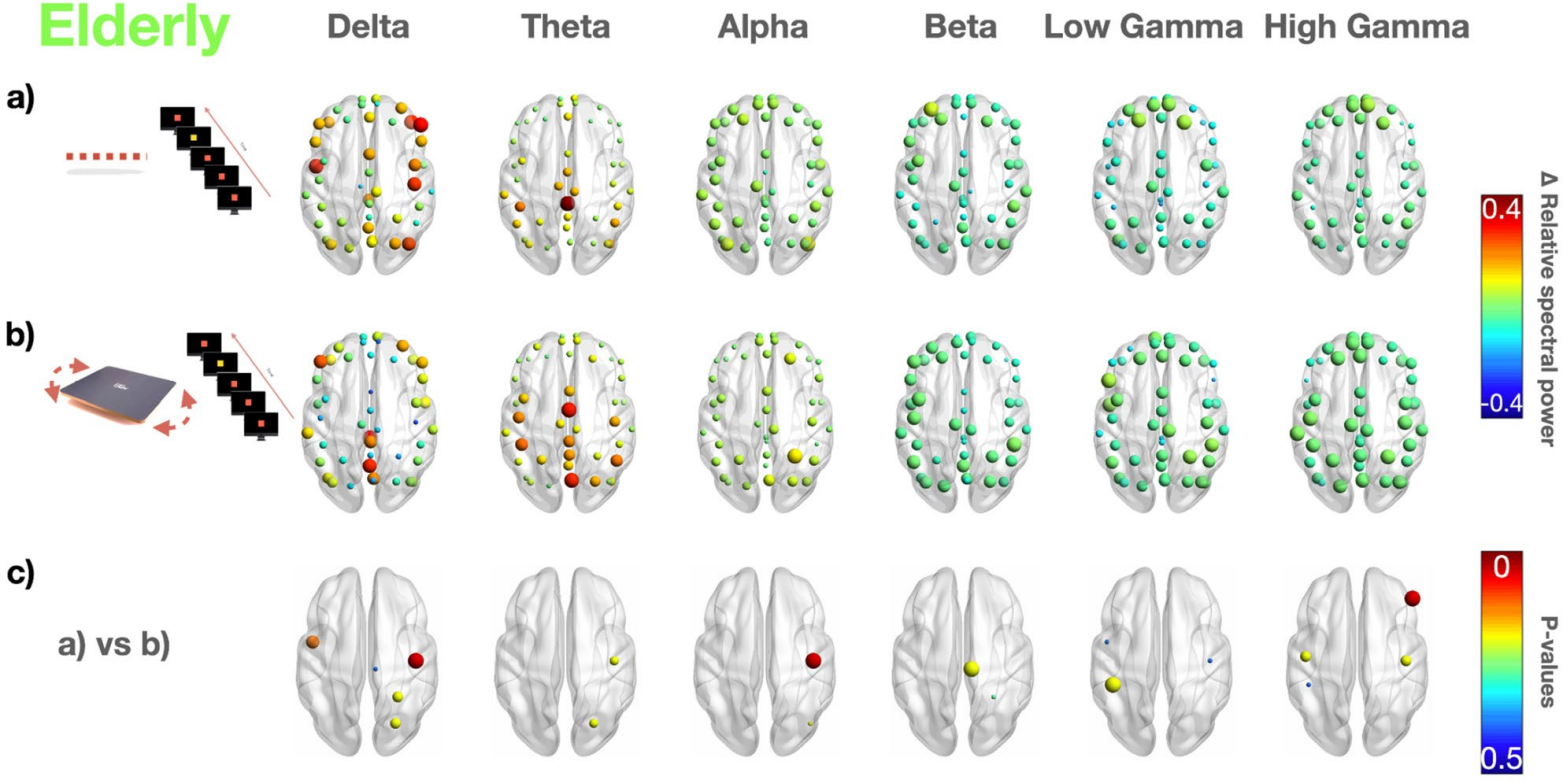
EEG power spectra during a dual-task static test (0%) and dynamic (core board) test in the elderly. Median values computed for the elderly for each ROI are represented by a sphere centered on the cortical region, whose radius is linearly related to power spectrum magnitude. Such information for panels (**a**) and (**b**) is also coded through a color scale titled “$\Delta$ relative spectral power”. We reported the difference ($\Delta$) of the power spectra between the 0.5 s-epoch post-odd stimulus and the 0.5 s-epoch pre-odd stimulus in the same postural conditions of Fig. 3. The p-values showing statistically significant differences between (**a**) and (**b**) condition are reported in (**c**). Each *P* < 0.05 is represented by a sphere centered on the cortical region, whose radius is linearly related to the magnitude, also coded using a color scale titled “P-values”.

**Figure 5:**
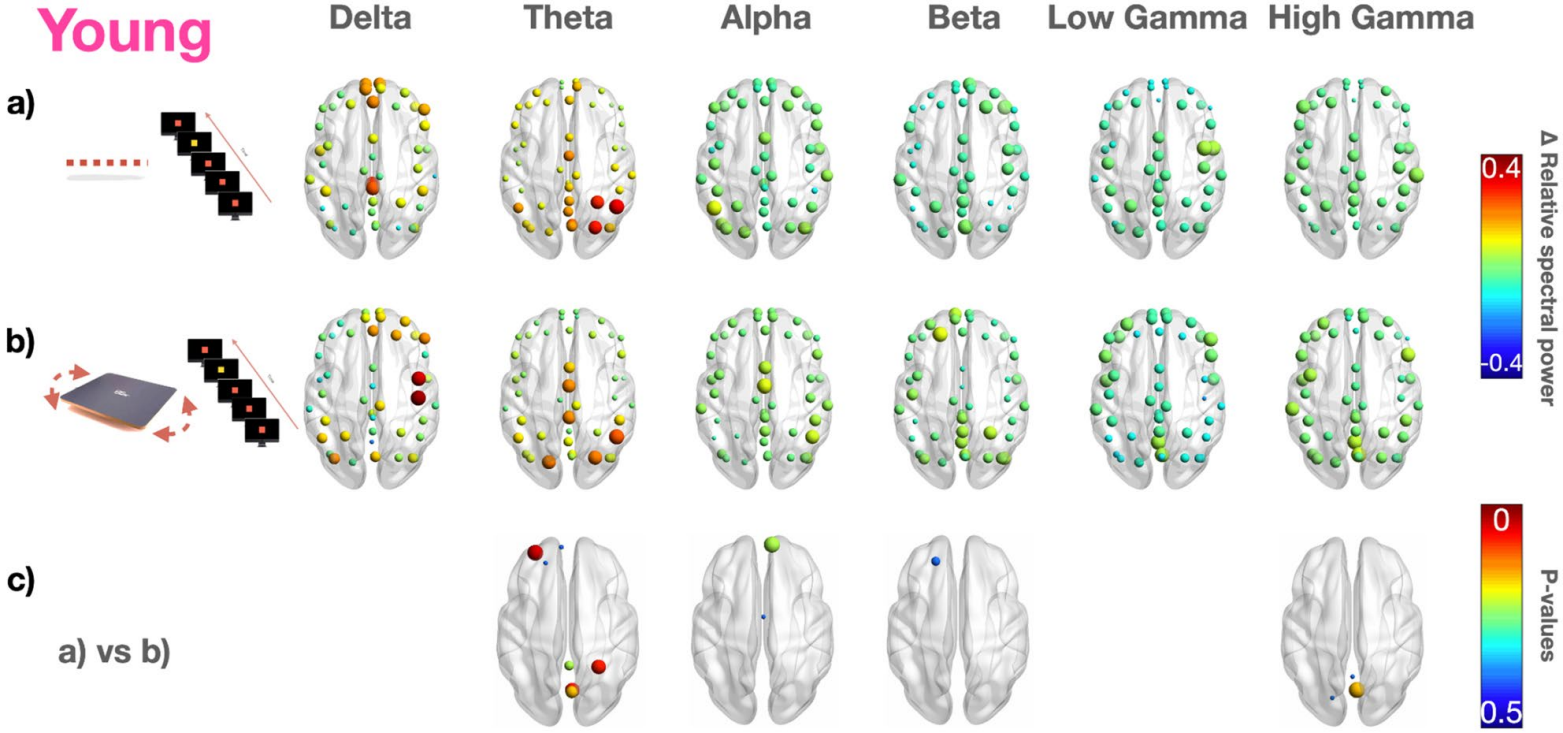
EEG power spectra during dual-task static (0%) and dynamic (core board) test conditions in younger adults. Median values computed among younger adults for each ROI represented by a sphere centered on the cortical region, whose radius is linearly related to power spectrum magnitude of the EEG data. Such information for panels a) and b) is also coded through a color scale titled “$\Delta$ relative spectral power”. We reported the difference ($\Delta$) of the power spectra between the 0.5 s-epoch post-odd stimulus and the 0.5 s-epoch pre-odd stimulus in the same postural conditions of Fig. 3. The p-values showing statistically significant differences between (**a**) and (**b**) condition are reported in (**c**). Each *P*-value < 0.05 is represented by a sphere centered on the cortical region, whose radius is linearly related to the magnitude of the EEG data, also coded using a color scale titled “*P*-values”.

Figures 4 and 5 report cortical activations during dual-tasking (i.e., mental counting of the odd stimuli during static and dynamic balance). All participants reported the right number of the odd stimuli, correctly performing the cognitive task.

Figures 4 and 5 report the difference of the power spectra between the 0.5 s-epoch post-odd stimulus and the 0.5 s-epoch pre-odd stimulus in the same postural conditions of Fig. 3. Statistically significant differences (p-values) are reported in Fig. 4c during static balance (simple standing) and in Fig. 5c during dynamic balance.

During static balance dual-tasking, the elderly showed (Fig. 4a) increased values of midline theta and showed higher values over frontal regions in high-gamma compared to younger adults (Fig. 5a). During dynamic balance dual-tasking, the elderly (Fig. 4b) increased their midline delta and high gamma compared to younger adults (Fig. 5b). Comparing cortical activations between static and dynamic tasks in the elderly, we observed significantly higher cortical activations localized over sensorimotor regions in the alpha and beta bands and over the frontal region in the high gamma during the dynamic balance test (Fig. 4c). A different pattern was observed in the younger adults, with an increase in the gamma band localized over the occipital regions during the dynamic balance test (compared to the static one) (Fig. 5c).

The delta power over the centro-parietal areas significantly increased during the dynamic balance test (compared to the static one) in the younger adults. When dual-tasking, the elderly showed a decrease in the delta rhythm in the centro-parietal area parallel to an increase in the theta rhythm in the occipital area during dynamic balance compared to static balance.

**Figure 6:**
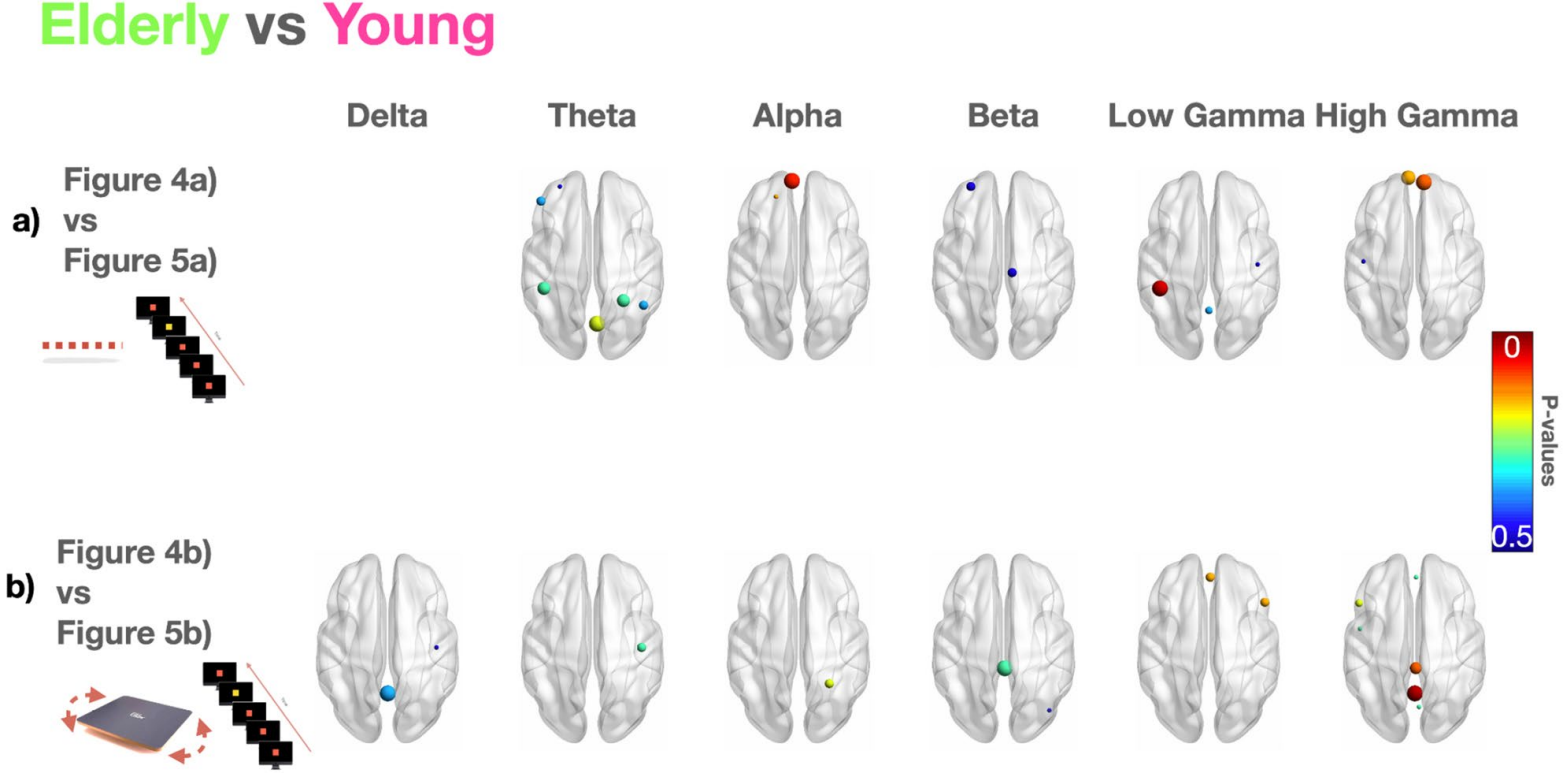
Statistics in the elderly vs. the younger adults during odd stimuli in static (0%) and dynamic (core board) balance tests. The *P*-values showing statistically significant differences between the younger adults and the elderly group during (**a**) visual odd stimuli and static balance (0%) and (**b**) during visual odd stimuli and dynamic balance (core board). Each *P*-value < 0.05 is represented by a sphere centered on the cortical region, whose radius is linearly related to the magnitude, also coded through a color scale titled “*P*-values”.

Comparing the performance (Fig. 6a,b) in dual-tasking in the elderly (Fig. 4) vs. the younger (Fig. 5) adults, we observed a significant increase in frontal and occipital theta rhythms and high frontal gamma in the elderly in dual-tasking in static balance (Fig. 6a).

**Figure 7:**
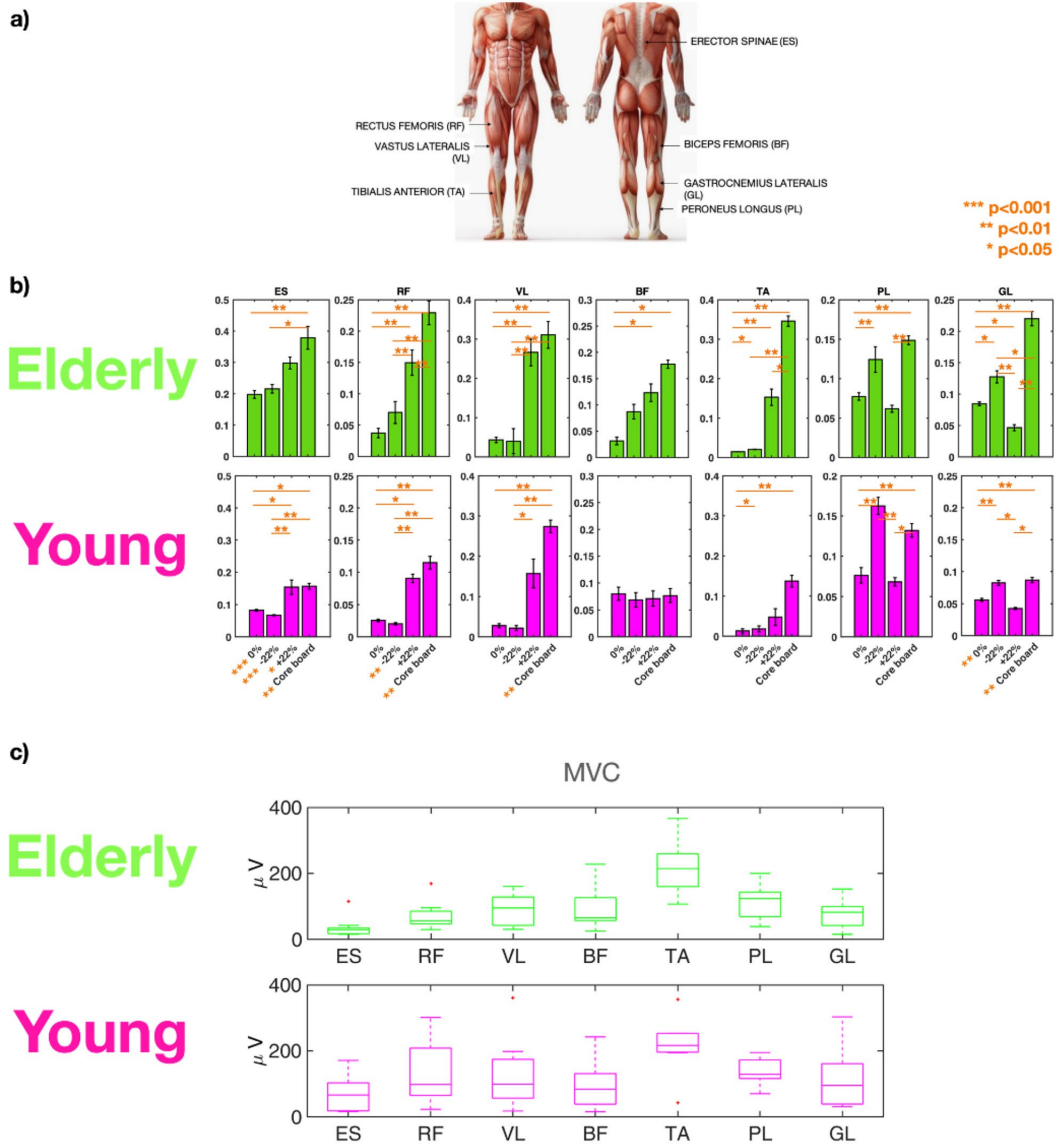
(**a**) Muscle activity as measured using EMG sensors.** b**) RMS EMG results. Median values across the elderly (in green) and the younger adults (in pink) of the RMS values for each muscle (ordered in column): Erector Spinae (ES), Rectus Femoris (RF), Vastus Lateralis (VL), Biceps Femoris (BF), Tibialis Anterior (TA), Peroneus Longus (PL) and Gastrocnemius Lateral head (GL) in static (0%, ± 22%) and dynamic (core board) conditions. P-values are represented by asterisks. In each box, p-values represent statistically significant differences among postural conditions within the same group. P-values reported in the x-axis represent statistically significant differences between the two groups under the same postural conditions. (**c**) MVC results. Boxplots display the maximum voluntary contraction values for the elderly (in green) and for the younger adults (in pink).

During dual-task static balance on the downward inclined plane (− 22%), the elderly showed higher cortical activations in the alpha and gamma bands over the occipital and frontal regions (compared to the younger adults). This pattern is similar to the one observed in the younger adults during the dynamic balance tests, suggesting a similar cognitive load in the elderly and the younger adults in these two different testing conditions. Higher cortical activation is observed in younger adults, during dual-task static balance on the inclined plane (+ 22%) over the frontal regions in the theta and low gamma bands, similar to the pattern observed during the static balance tests. During the same task, the elderly show higher activation on the frontal and central regions in the beta band.

Results of all the statistical tests for the EEG power spectra are provided in Supplementary Materials.

In Fig. 7b, we reported the RMS values of the muscular activation signals normalized by the median RMS value of the maximum voluntary contractions across subjects of the same group (Fig. 7c) for: Erector Spinae (ES); Rectus Femoris (RF); Vastus Lateralis (VL); Biceps Femoris (BF); Tibialis Anterior (TA); Peroneus Longus (PL) and Gastrocnemius Lateral head (GL). Each barplot shows the median values computed for the elderly (first row, in green, of Fig. 7) and the younger adults (second row, in pink, of Fig. 7).

RMS values for the ES significantly differ in all the postural conditions between the two groups: the elderly showed higher values and an increasing trend from 0% inclined surface to the core board (first column of Fig. 7). The RMS values for the RF were significantly higher in the elderly on the − 22% inclined surface and on the core board (*P* < 0.01). The RMS of the GL was significantly higher in the elderly on the 0% plane and on the core-board (*P* < 0.01).

The PL and GL activity in the − 22% condition revealed a plantar-flexion action, similar in the two groups, complemented by two different knee strategies. The elderly showed higher RF and BF activation, whereas the younger adults displayed a preferential recruitment of knee flexors.

Results obtained for the + 22% condition suggested a different ankle strategy to keep balance: the elderly showed higher activation of TA, PL and GL, with the higher difference in the recruitment of TA. Both groups had higher activity of the knee extensors than of the BF.

The task on the core board highlighted a further activation of the plantarflexor and dorsiflexor muscles of the ankle joint in the elderly (i.e., higher activation of TA, PL and GL) than in the younger adults, who appeared to prioritize the recruitment of the PL for fine movement control in the medial-lateral plane^38^. The activity of the RF, which serves both as knee extensor and hip flexor, was also found to be significantly higher in the elderly than in the younger adults on the core board.

No significant differences were found comparing EMG data during the postural task with and without the visual oddball. We obtained the same RMS trend reported in Fig. 7 also without normalization (see Supplementary Materials).

## Discussion

Our findings provide evidence of different motor control strategies to maintain static and dynamic balance in the elderly compared to younger healthy adults.

During the static balance test, the power in the delta band over the frontal regions was higher in the elderly. Younger participants had higher activation on frontal and occipital areas of the fast frequencies (i.e., alpha, beta and gamma bands). Both groups showed mid-line fronto-central theta rhythms, a neural signature of focused attention^39^, e.g., the attention-based process of maintaining posture. During the dynamic balance test, the theta rhythms over the sensorimotor and occipital areas of the elderly increased. When adding a visual oddball task, the elderly showed a more robust activation over the sensorimotor areas during the static balance test compared to the single-task condition. Dynamic balance tests showed similar findings, but magnified. In the younger group, the static balance tests during dual-tasking were associated with occipital theta and gamma rhythm, and increased frontal alpha, which again are even more prominent during the dynamic balance tests.

The different cortical activation strategies are more evident during the dual-tasks, but they already emerged during the quiet static balance tests. The elderly showed high delta activation over the frontal areas, a process hypothesized to contribute to maintaining postural control^21,22^. The younger adults showed prevalent fast frequencies (i.e., beta and gamma bands) over the frontal and occipital areas. This activation over the occipital lobes may suggest an ongoing attention-based task to maintain upright posture supported by a visual fixation strategy in this group.

As represented in Fig. 2, in the younger adults, all variables quantifying body sway (i.e., ROM-AP, ROM-ML, SP, CEA, ROM-VT) showed higher interquartile range during the single-task compared to the dual-task condition. Focused attention on external elements (such as staring at a black screen - i.e., our single-task) is known to lead to reductions in postural sway^40^, which is further reduced when adding a cognitive task, such as counting the odd stimuli (i.e., dual-task)^41^. The same result was obtained for the elderly in all postural tasks, except for the core board. With the postural task becoming more demanding (i.e. dynamic postural task), the balance performance deteriorates in the elderly from the single- to the dual-task, showing similar median values for all variables with more significant variability.

Dual-task is a well-known strategy to increase the cognitive load in experimental conditions and mimics real-life settings in which balance control is coupled with tasks such as speaking or texting. A standard paradigm used to simulate real-life is the visual oddball, during which the participant is asked to count the stimuli presented on a screen that differ from the more common one. In our experiment, the dual-task addressed the issue of the competing cognitive resources the elderly deploy during the stance test. In line with previous research^2,23^, we expected an increase of cortical recruitment to keep up performance in the elderly. The observed activation of the posterior areas in the theta band supports this hypothesis: it is coupled with a recruitment of sensorimotor cortices, confirmed by the muscle activation patterns hinting to an increased muscle activity to maintain posture.

In younger adults, we observed that during dual-task dynamic postural tests, the strong activation of the occipital and frontal areas (in beta and gamma bands) was consistent with the oddball task. A limitation of the study is the lack of a co-registered electrooculogram and eye tracking to ensure gaze fixation. While this may not have been an issue during the cognitive task, when target fixation is required to ensure correct execution, a wandering gaze may arise when idling. We obtained similar activation over the occipital lobes (in beta and gamma bands) during quiet standing in the younger adults. These findings suggest that the underlying neural circuits may be similar, but likely they are used with a different level of efficiency. The higher power in beta and gamma bands over the occipital cortex in the younger adults may point to a different processing strength between the two populations, with younger participants processing visual cues more efficiently, as already reported for orientation discrimination or contrast detection^42–45^. This difference may also be reported to the greater difficulty the elderly display in adapting to rapidly changing environmental conditions^38^.

The main differences in cortical activations between the two groups appear during dual-tasking and suggest that the elderly needed to recruit adaptive reserves. This finding contrasts with previous work^46^ which described increased cortical activation during dual-tasking indicating a defective compensatory mechanism in the elderly. It is also possible that the different nature of the task – static vs. dynamic – may make these findings not directly comparable.

In normal aging, structural and functional physiological brain changes occur. Structural changes (i.e., cortical thinning, local brain atrophy, etc.) occur together with functional maladaptive brain activity, namely decreased functional specificity and loss of functional lateralization. These phenomena trigger the activation of compensatory mechanisms^47^. This has been referred to as compensatory cognitive scaffolding, whose efficacy largely depends on each individual’s cognitive reserve^48^. This model explains older individual’s level of cognitive functioning: this relies on compensatory mechanisms by which supplementary higher level cortical networks are recruited to accomplish tasks supposedly be highly automatized, as the control of upright stance^49^. The increased allocation of attention resources to handle a challenging postural task, together with a cognitive oddball paradigm, led the older group to an increased recruitment of cortical areas. This is not needed in younger adults who rely on more automatized and less central processes, having more cognitive resources available to accomplish the task^50,51^.

The elderly showed higher muscular activity. The higher activation of the ankle plantarflexor and dorsiflexor muscles in the elderly suggests a different neuromuscular strategy. A possible stiffening action at the ankle joint in static balance becomes more evident during the dynamic tasks. The larger body sway observed in the elderly during the static and dynamic tests supports such hypothesis. Simultaneous contraction of both agonist and antagonistic muscles (i.e., joint stiffening^50–52^) was associated with increased postural sway and risk of falls in the elderly^53–55^. Joint stiffening is a maladaptive strategy to reduce postural sway, as a reduction in flexibility hampers an adaptive response to perturbation^56,57^. In both groups, PL shows a strong activation during the static balance test due to greater stabilization needs in the medio-lateral direction^58^ which shows larger range of motion of body sway than in the antero-posterior direction, especially in dynamic conditions. The elderly augment the PL activity concurrently activating TA and GL. Younger adults preferentially control the ankle joint relying on the PL activity for fine movement control in the medio-lateral plane^38^.

These mechanisms are complemented with a higher level of activation of the knee extensors (RF and VL) and flexors (BF) during the dynamic balance in the elderly, most likely to add stiffness to the knee. The younger adults prefer lowering their center of mass to increase their stability. Besides not being significantly different, the Center of Mass (CoM) range of motion in the vertical direction was lower and less variable during the dynamic task in the younger adults than in the elderly. This supports the conclusion that the elderly implement a less efficient balance control strategy.

RMS values for the ES significantly differ in all the postural conditions between the two groups: the elderly show higher values and an increasing trend from 0% inclined surface to the core board (first column of Fig. 7). The RF activity, which serves as knee extensor and hip flexor, was also significantly higher in the elderly during the dynamic balance tests and associated with a significantly higher level of ES activity.

Our findings indicate that the elderly need to increase cortical recruitment during challenging postural tasks with additional cognitive load. Dynamic postural control during dual task tests requires the involvement of the whole sensory-motor network: in the elderly this reflects in activating muscles that stiffen the lower limbs and trunk. Younger subjects are apparently undisturbed by the dual-tasking, with a possible visual fixation strategy to maintain dynamic balance, which can only be inferred from our data. Their motor strategy seems more efficient and less energetically demanding, given the overall lower contraction levels and reduced number of involved muscles.

These observations may guide the design of future rehabilitative interventions, customized based on the preferential postural strategy. These findings will also provide insight into the neural processes underlying postural control.

## Methods

We recorded EEG scalp brain activity using a high-density (i.e., 256 electrodes) EEG system. EMG data were simultaneously recorded using 8 wireless probes that we positioned on relevant muscles of the leg and the trunk. Subsequently, we computed: 1) for each region of interest in the brain cortex, the EEG source waveform in the time domain and its spectral power distribution in the canonical EEG frequency bands and 2) the root mean square values of the EMG data collected during various postural tasks (i.e., standing on a 0%, + 22%, − 22% inclined planes and on a core board) and dual postural-visual tasks (adding a visual oddball – i.e., the participant is asked to count the odd stimuli presented on a screen that differ from the more frequent stimuli – to the previous conditions) in nine healthy elderly ($> 64 \,\hbox {y}$) and eight younger ($< 35\,\hbox {y}$) adults.

We identified the power spectral content of the derived EEG source generators of brain regions that were active during different postural testing conditions and dual-tasks in the cohort of young and old healthy adults. We further estimated the EEG spectral power and EMG amplitude differences between the two groups.

### Participants

Nine healthy right-handed elderly ([64-76] y) and eight healthy right-handed younger ([24-34] y) adults were recruited. Elderly were referred to as individuals 65+ years of age, according to the United Nations definition. The age range for selecting the younger adults was chosen by considering that strength and physical performance reach their peak between 18 and 39 years of age^59,60^.

Exclusion Criteria were: (1) Diagnosis of neuropathy or sensation of tingling in the legs or feeling of having reduced sensitivity to feet or legs; (2) Diabetes mellitus; (3) Any rheumatological pathology; (4) Orthopedic problems (e.g., arthrosis); (5) History of fractures of the lower limbs or feet; (6) History of spinal surgery; (7) History of hip and/or knee replacement surgery; (8) History of stroke even if completely recovered. Hypertension was not considered as a contraindication if under drug treatment and with good control.

This study was carried out in accordance with the recommendations of the Ethics Committee of the Teaching Hospital of Padua (n.AOP2025). All subjects signed written informed consent.

### Behavioral tests

Participants were evaluated using the Trail Making Test, i.e., a neuropsychological test in two parts, to ensure that elderly participants did not have any movement speed and visual attention deficits. In part A, i.e., TMT-A, the subject’s task is to quickly draw lines on a page connecting 25 consecutive numbers without lifting the pen from the paper. In case errors occur, participants are alerted, and correction is allowed. The performance is assessed using the time needed to complete the trial correctly. Part B (i.e., TMT-B) tests how fast the participant can connect numbers and letters in alternating increasing sequence (i.e., 1-A-2-B, etc.). Part B is more challenging than Part A because it is a more difficult cognitive task requiring movement speed and visual search^61^. The TMT-B evaluates visual attention, movement speed, and cognitive impairments.

To exclude participants performing under the threshold of normality based on normative values computed by taking into account each subject’s education level and age^34^, education was assessed as asking the number of years in school.

None of the 9 elderly participants in the study had deficits in movement speed or visual attention, as verified by TMT scores within a normative threshold considering age and education^34^.

Lilliefors test^62^ was used to test the null hypothesis that data come from a normally distributed population.

### Data acquisition

High-density EEG recordings were acquired at Padova Neuroscience Center inside a dimly lit sound-attenuated and electrically shielded room using the Geodesic Sensor Net system with 256 electrodes (Electrical Geodesic Inc., Eugene, OR, USA). Electrode-skin impedances were maintained $< 40~k \Omega$. The recordings were sampled at 500 Hz, referenced to Cz. In parallel, a system consisting of EMG probes and Inertial Measurement Units (IMU - Cometa Srl, Milan, Italy) was used to record muscle activity signals and kinematic data synchronized with EEG recordings. EMG recordings were acquired from the following muscles in the right leg (Fig. 7a): Rectus Femoris (RF), Vastus Lateralis (VL), Tibialis Anterior (TA), Peroneus Longus (PL), Gastrocnemius Lateral head (GL), Biceps Femoris (BF); and from Erector Spinae (ES) with Cometa MiniWave Waterproof EMG sensors (Cometa srl, Milan, Italy). An expert researcher selected the position of the electrodes to minimize crosstalk effects^63^ as suggested by^64,65^. Skin preparation was performed by removing dead skin cells (i.e., gently scrubbing) and moisturizing the skin if participants had a dry skin, or rubbing it with alcoholic wipes when they had an oily skin^65^. The electrode-skin impedance was maintained $< 74~k\Omega$. The recordings were sampled at 2 kHz.

One IMU was secured on the fifth lumbar vertebra (L5) with a double-sided adhesive tape to measure the the pelvis’s movements and thus to estimate the kinematics of the body CoM^66–69^. The IMU consisted of a 3D accelerometer, a 3D gyroscope and a 3D magnetometer. For the purpose of this study, only the 3D accelerometer data were collected and sampled at 142.85 Hz.

Synchronization of EEG and EMG-IMU recordings was assured by physically connecting the TTL trigger out channel of the EMG-IMU receiver to the “synch-box” of the EEG system. A TTL is an electrical signal based on the transistor-transistor logic, which produces a two-level signal: a low voltage level (from 0 to 0.8 V) and a high voltage level (normally ranging between 2 and 5 V). At the stop recording command on the EMG-IMU system, the receiver switches the TTL sent to the to the EEG synch-box from the low to the high level, and a time stamp was saved.

### Experimental protocol

*Baseline*. Three minutes of quiet upright standing (without shoes) were collected at the beginning and at the end of the experiment. Participants were instructed to keep their feet parallel and at shoulder width, while staring at a black screen.

*Static postural tests*. Participants were standing upright without shoes on level ground or on an inclined surface at different angles. The feet position was kept consistent across trials. For the + 22% condition, the subject was instructed to stand on an inclined surface at + 12°. Thus, the participants had dorsiflexed ankle and toes pointing upward. For the − 22% condition, the fixed plane was inclined at − 12° and the participants had ankles in plantarflexion and the toes pointing downward. For the 0% condition, participants were standing upright on the floor.

#### Dynamic postural tests

 In this experimental condition, we used a “rocker” balance board with a flat and rectangular shape and a semi-cylindrical base. Due to its semi-cylindrical stationary base, it has a range of motion limited to one axis (anteroposterior or medio-lateral depending on how the board is positioned). Participants placed their feet parallel and at shoulder width and the core board could only rock in the antero-posterior direction. Specifically, participants were asked to keep balance on the 50 cm x 50 cm 1 degree-of-freedom wooden core board with a total of $24 ^{\circ }~(\pm 12 ^{\circ })$ front-to-back tilt.

Both the static and dynamic postural tasks were tested with and without the cognitive perturbation, i.e., a visual oddball, as a dual task.

*Visual oddball*. Participants were standing in front of a black screen and instructed to stare at it. The distance between the participant and the screen was kept constant across trials and participants (i.e., 1 m). After 30 s, the presentation (block) of a sequence of repetitive stimuli (i.e., a 3 cm^2^, 3 cm^2^ red square in the center of the black screen) was infrequently interrupted by a deviant stimulus (i.e., a 3 cm^2^ yellow square in the center of the black screen). Each stimulus lasted 500 ms. The inter-stimulus interval (measured from the disappearance of the previous stimulus – i.e., stimulus offset – and to the appearance of the following one – i.e., stimulus onset) was randomly set from 500 ms to 1 s.

Each block contained circa 80 repetitive stimuli, and circa 20 deviant stimuli, for a total of 100 stimuli per block. The whole experiment included four blocks (3 for the static postural conditions, i.e., 0% + 22% and − 22%, and one for the dynamic postural condition, i.e., the core board experiments) performed in a pseudo-randomized order. Each block lasted about 3 minutes. Participants were asked to count the deviant stimuli, i.e., the yellow squares, randomly shown on the screen. Short breaks were taken when needed.

### Data processing

*EEG processing*. EEG data were zero-phase-high-pass-filtered above 1 Hz through a 4th order Butterworth filter and then zero-phase-low-pass-filtered below 70 Hz through a 4th order Butterworth filter avoiding phase distortion. Channels in the cheeks and in the neck were discarded (204 channels left). EEG epochs were then extracted from the continuous dataset and time-locked from -500 to 500 ms relative to the onset of each image during the visual oddball. The 30-s recorded before and after the dual-task were divided into non-overlapping epochs of 1 s. Noisy channels were identified by visual inspection and interpolated using the nearest-neighbor spline method (average percentage of channels interpolated: 1.5%). Individual epochs containing non-stereotyped artifacts, peri-stimulus eye blinks and eye movements (occurring within $\pm 500 \,\hbox {ms}$ from stimulus onset) were also identified by visual inspection and removed from further analysis (average percentage of epochs removed: 10%). Data were cleaned from remaining physiological artifacts (eye blinks, horizontal and vertical eye movements, muscle potentials and other artifacts) through a Principal Component Analysis (PCA)-informed Independent Component Analysis (ICA) algorithm implemented in EEGLAB (average percentage of components removed: 9.1%).

We applied the LAURA algorithm implemented in Cartool^70^ to compute the source reconstruction^71,72^ taking into account the patient’s age to calibrate the skull conductivity^73–75^. The method restricts the solution space to the gray matter of the brain. Then, the cortex was parcellated into 45 brain regions of interest (ROIs)^76^: *(Right and Left:) Precentral gyrus; Superior frontal gyrus, dorsolateral; Superior frontal gyrus, orbital part; Inferior frontal gyrus, opercular part; Inferior frontal gyrus, triangular part; Inferior frontal gyrus, orbital part; Rolandic operculum; Superior frontal gyrus, medial; Superior frontal gyrus, medial orbital; Superior occipital gyrus; Middle occipital gyrus; Inferior occipital gyrus; Postcentral gyrus; Superior parietal gyrus; Inferior parietal, but supramarginal and angular gyri; Supramarginal gyrus; Angular gyrus; Paracentral lobule* and *Occipito-temporal gyrus; Supplementary motor area; Median cingulate and paracingulate gyri; Posterior cingulate gyrus; Calcarine fissure and surrounding cortex; Anterior cingulate and paracingulate gyri; Cuneus; Lingual gyrus; Precuneus*.

The dipoles in each ROI were represented with one unique time-series by applying a singular-value decomposition^77^.

For each time-series that represented the activity in each ROI, we computed the absolute and the relative power spectra in the canonical EEG frequency bands, i.e., delta [1-4] Hz, theta ]4-8] Hz, alpha ]8-12] Hz, beta [14-24] Hz, low gamma [30-50] Hz, high gamma ]50-70] Hz. Each 1-s EEG source-waveform epoch was multiplied by a window function, i.e., Hamming window. The periodograms of all modified epochs referred to the same experimental condition were then averaged to estimate the power spectral density of each condition. The area under the curve (representing the power spectral density as a function of the frequency) was estimated for each EEG frequency band. The power of each EEG frequency band was divided by the power in the interval [1-70] Hz to compute the relative power.

Lastly, we performed two-sided Wilcoxon rank sum tests on the relative power spectra values for each ROI under the same postural/dual-task condition between the two groups to test for significantly different activations between the elderly and the younger adults. We performed two-sided paired samples Wilcoxon signed-rank tests inside the same group to compare relative power spectra values for each ROI between the different postural/dual-task conditions. As input of the statistical tests, we used either the relative power spectral values during the baseline condition (i.e., simply standing in front of a black screen) or the difference between the relative power spectral value post-stimulus (i.e., 500 ms after the stimulus onset) and pre-stimulus (i.e., 500 ms before the stimulus onset) for the odd and frequent stimuli. Since the event-related phenomena represent frequency-specific changes of the EEG activity and consist of decreases or increases of power in given frequency bands; this pattern is meaningful only if compared to a baseline measured before the event^78^.

*EMG processing* EMG data (time-locked with the EEG recordings) were zero-phase-filtered in the interval [20-250] Hz through a 4th-order Butterworth filter^79^. EMG segments containing artifacts were identified using a threshold ($|EMG| > 5*standard~deviation$) and removed from further analyses (percentage of data-points removed: < 3.5% for the elderly; < 3% for the younger adults).

We computed the Root Mean Square (RMS) value for each EMG signal. In order to compare the muscular activity between the two groups (i.e., elderly and younger adults), the RMS of each muscle activation during each task was divided by the within-group median RMS value of the same muscle while performing a Maximum Voluntary Contraction (MVC). Due to the experimental setup constraints, MVCs were performed with the participants standing in the shielded room and equipped with the EEG cap. Not all the participants were able to perform a proper MVC and normalizing the RMS within-subjects would lead to unreliable results. However, RMS obtained from each muscle and within groups showed low variability. Hence, we assumed that median values are representative of the muscular activity of the elderly and the younger adults.

We then compared the normalized RMS value of each muscle between the two groups: two-sided Wilcoxon rank sum tests were applied under the same postural condition (i.e., 0%, $\pm 22\%$, core board); paired samples two-sided Wilcoxon signed rank tests were applied among all the four different postural conditions within each muscle and group.

*IMU processing*. To estimate each participant’s body sway, L5-acceleration from all the collected trials was first low-pass filtered with a zero-lag 2nd-order Butterworth filter (cutoff 15 Hz)^80^. A roll-pitch correction was then estimated from the baseline condition to correct for possible misalignment of the sensor axes with the global anterior-posterior (AP) and medial-lateral (ML) directions of the body^81^. This correction was eventually applied to the acceleration signals gathered from postural tasks (static and dynamic). For the dynamic postural task only (i.e., postural task on the core board), we also estimated the vertical component (VT) of the acceleration.

In order to estimate the Center of Mass (CoM) trajectory, a strap-down double integration was then applied to the corrected acceleration data^80^. After each integration step, a high-pass 1st-order Butterworth filter (cutoff 0.2 Hz for AP and ML directions, 0.5 Hz for VT direction) was used to account for possible drift effects of the integral. Lastly, the double integrated signal was low-pass filtered with a 5th-order Butterworth (cutoff 10 Hz). This method provides a reliable estimate of the Center of Mass trajectory of the human body^82^. Windows of 30 s of the estimated CoM trajectory were then retained for further analysis from the baseline and visual oddball conditions of each postural task. The range of motion in the AP and ML directions (ROM-AP and ROM-ML, respectively), the Sway Path length (SP) and the area of the ellipse containing the 95% of the CoM trajectory (CEA) were computed on the AP-ML plane^83^. The range of motion in VT direction (ROM-VT) was computed to estimate each participant’s body sway for the core board postural task.

The estimated parameters were then normalized for the participants’ height and body mass via a detrending normalization technique^84,85^. In brief, the correlation between each estimated parameter and subjects’ anthropometry was tested. Then, parameters values were iteratively corrected via the linear model that best interpolates the data. As the last step, detrended data were corrected by the mean value of the estimated parameters. This method has the advantage to keep values with their original range and measurement unit, but it removes any dependencies from confounding variables^84^.

For all variables, within-group differences between single-task and dual-task conditions for both the elderly and the younger adults, and the between-group differences in each postural condition were tested via paired and unpaired samples two-sided Wilcoxon tests (p = 0.05), respectively. We then compared each variable among the four postural conditions within-group for both single and dual-task via a paired samples two-sided Wilcoxon test ($\hbox {p} = 0.05$).

Considering the sample size and the exploratory nature of the study, no corrections for multiple comparisons were performed in hypothesis testing.

### Ethics statement

All participants gave their written informed consent prior to the experiment and the study received the approval of the Ethics Committee of the Teaching Hospital of Padua, Padova, Italy (n.AOP2025). All experiments of this study were performed in accordance with relevant guidelines and regulations.

## Supplementary Information

Supplementary material 1 (xlsx 3673 KB)
